# Diels-Alder Additions as Mechanistic Probes–Interception of Silyl-Isoindenes: Organometallic Derivatives of Polyphenylated Cycloheptatrienes and Related Seven-Membered Rings

**DOI:** 10.3390/molecules25204730

**Published:** 2020-10-15

**Authors:** Michael J. McGlinchey

**Affiliations:** School of Chemistry, University College Dublin, D04 V1W8 Belfield, Dublin 4, Ireland; michael.mcglinchey@ucd.ie

**Keywords:** isoindenes, tetracyanoethylene, ferrocenylhexaphenylcycloheptatriene, tropylium ions, hexanaphthylbenzene, X-ray crystallography, organometallic molecular brake

## Abstract

The intermediacy of short-lived isoindenes, generated in the course of metallotropic or silatropic shifts over the indene skeleton, can be shown by Diels-Alder trapping with tetracyanoethylene, leading to the complete elucidation of the dynamic behaviour of a series of polyindenylsilanes. Cyclopentadienones, bearing ferrocenyl and multiple phenyl or naphthyl substituents undergo [4 + 2] cycloadditions with diaryl acetylenes or triphenylcyclopropene to form the corresponding polyarylbenzenes or cycloheptatrienes. The heptaphenyltropylium cation, [C_7_Ph_7_^+^], was shown to adopt a nonplanar shallow boat conformation. In contrast, the attempted Diels-Alder reaction of tetracyclone and phenethynylfluorene yielded electroluminescent tetracenes. Finally, benzyne addition to 9-(2-indenyl)anthracene, and subsequent incorporation of a range of organometallic fragments, led to development of an organometallic molecular brake.

## 1. Introduction

While Diels-Alder cycloadditions have been the cornerstone of much elegant natural product chemistry, as in the first total synthesis of a steroid (cortisone by Woodward [1]) or in the preparation of molecules having unprecedented symmetry such as cubane by Eaton [2], triquinacene by Paquette [3], and cubic graphite by Pascal [4], or exhibiting novel materials properties as in Mullen’s superacenes [5], they can also play a role in the elucidation of reaction mechanisms. Herein, we discuss the use of Diels-Alder reactions to trap proposed short-lived intermediates, to provide convenient high yield routes to sterically crowded organic and organometallic molecules and, even when they do not proceed as anticipated, to lead to unexpected novel products. 

## 2. Diels-Alder Trapping of Isoindenes

### 2.1. Metallotropic Shifts

**Scheme 1:**
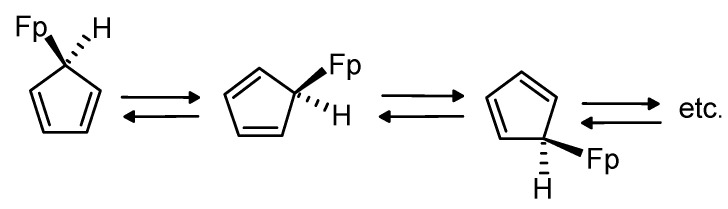
Metallotropic shifts around a cyclopentadiene ring, where Fp = (C_5_H_5_)Fe(CO)_2_.

In the first example, we consider one of the now classic studies of fluxional behaviour in organometallic chemistry. The preparation of bis(cyclopentadienyl)dicarbonyliron, (C_5_H_5_)_2_Fe(CO)_2_, raised a fascinating mechanistic observation: X-ray crystallography revealed that while one of the cyclopentadienyl rings was η^5^-bonded, in the other ring only a single carbon was attached to the iron atom. Moreover, although this latter fragment exhibited ^1^H NMR signals in the ratio 1:2:2 at low temperature, these peaks coalesced as the temperature was raised [6]. It was eventually established that the (η^5^-C_5_H_5_)FeCO)_2_ (Fp) moiety was undergoing a series of [1,2] migrations (subsequently reformulated in Woodward-Hoffmann terms as [1,5]-suprafacial sigmatropic shifts), as shown in Scheme 1. 

**Scheme 2:**
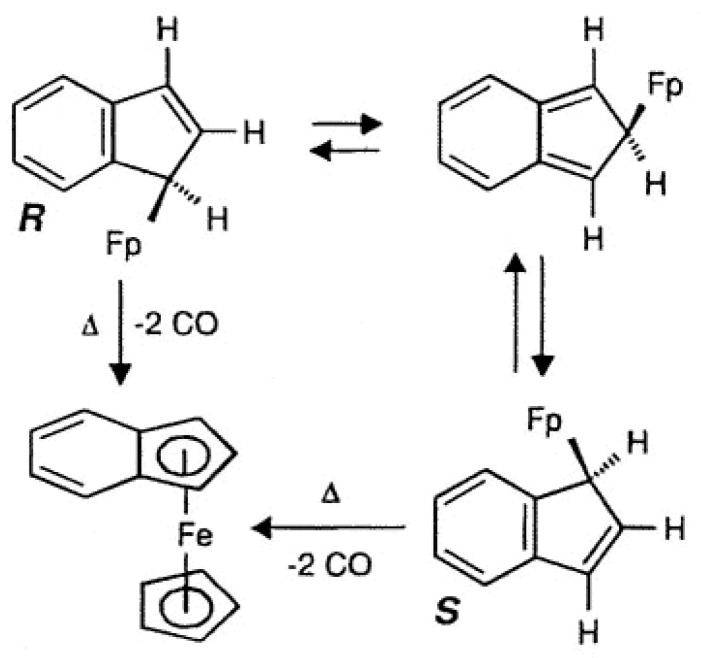
Consecutive [1,5] metallotropic shifts leading to interconversion of enantiomers.

However, when one ligand was replaced by an indenyl substituent, as in Scheme 2, a potential problem arose. If such a [1,5] shift were to occur, this would involve the intermediacy of an isoindene with partial disruption of aromaticity and, presumably, a substantially increased barrier to migration. This question is not resolvable by simply raising the temperature to monitor peak coalescence between the H(1) and H(3) sites because, already at 70 °C, there is evident decomposition with loss of the carbonyl ligands and formation of benzoferrocene [7,8]. 

**Figure 1:**
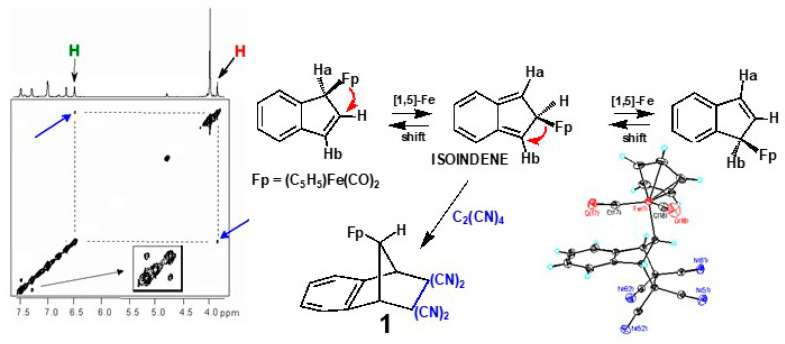
(**Left**) 500 MHz 2D EXSY NMR spectrum showing exchange between **Ha** and **Hb** in (η^5^-C_5_H_5_)(CO)_2_Fe(η^1^-C_9_H_7_) at 45 °C, and (**right**) molecular structure of **1**, the Diels-Alder adduct of tetracyanoethylene (TCNE) to the isoindene intermediate.

We chose to reinvestigate this system taking advantage of the development of the 2D EXSY NMR technique that facilitates the observation of chemical exchange processes without the need to raise the temperature such that line broadening is evident. This approach is ideal for temperature-sensitive molecules in which the barrier to exchange is high. The spectrum shown in Figure 1 clearly reveals exchange of the sp^3^-bonded and sp^2^-bonded protons–H(1) and H(3), respectively, and the barrier was evaluated as ~85 kJ mol^−1^ [9], markedly higher than the 45 kJ mol^−1^ value previously found for migration round the cyclopentadienyl ring in (η^5^-C_5_H_5_)Fe(CO)_2_(η^1^-C_5_H_5_).

Conclusive evidence for the intermediacy of the isoindene was provided by its reaction with tetracyanoethylene (TCNE) at room temperature to form the Diels-Alder adduct, **1**, that was unambiguously characterised by X-ray crystallography (Figure 1) [9], even though it had been suggested that the absence of an available diene unit would render the system incapable of participating in a [4 + 2] cycloaddition [10].

### 2.2. Silatropic Shifts

**Figure 2:**
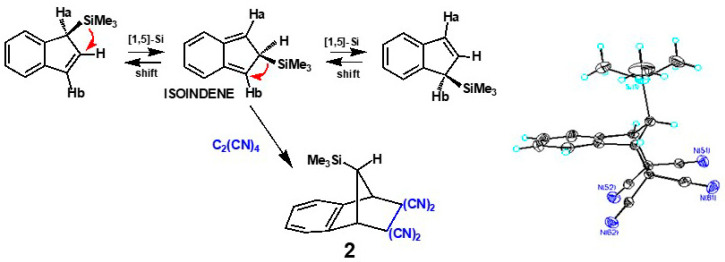
Molecular structure of **2**, the Diels-Alder adduct of TCNE to the isoindene intermediate derived from 1-trimethylsilylindene.

In closely related systems, the [1,5]-migration of a trimethylsilyl (germyl or stannyl) substituent around the indenyl ring was investigated, and the intermediate isoindenes were trapped by Diels-Alder reaction with maleic anhydride or TCNE [11,12,13,14], as depicted in Figure 2. 

**Scheme 3:**
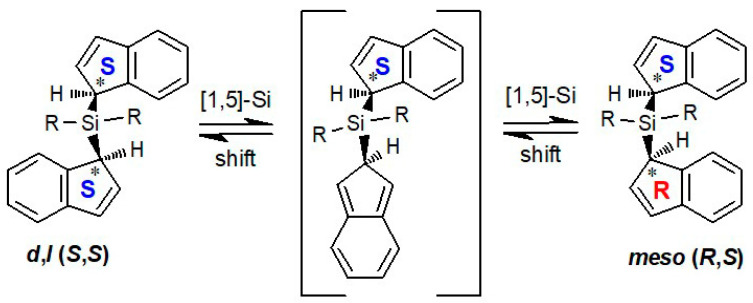
Interconversion of diastereomers of bis(indenyl)dimethylsilane.

In bis(indenyl)dimethylsilane, interconversion of the meso and dl isomers was monitored by following the NMR behaviour of the methyl groups (Scheme 3). In the mirror-symmetric meso isomer they are nonequivalent, whereas in the dl case the C_2_ symmetry renders the methyls equivalent. Again, the intermediate isoindene was trapped as its Diels-Alder TCNE adduct [15]. 

**Scheme 4:**
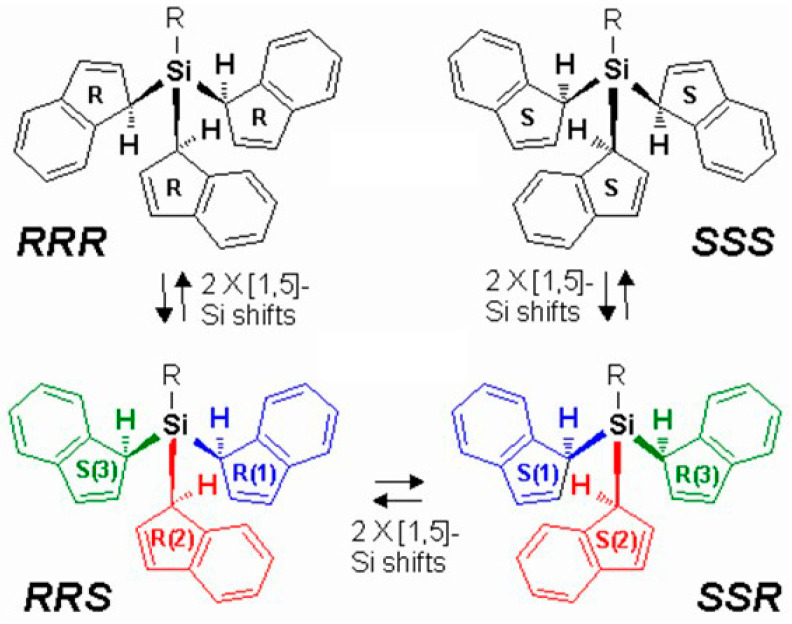
Interconversions of the eight different indenyl ring environments of tris(indenyl)methylsilane, **3**. The configurational inversion of a single indenyl ring requires two [1,5]-suprafacial sigmatropic shifts [17].

This work has been extended to bis-, tris- and tetrakis-indenyl systems [16,17]. A particularly dramatic case is tris(indenyl)methylsilane, **3**, which gives rise to *RRR*, *RRS*, *RSS* and *SSS* isomers in a 1:3:3:1 ratio, where the *R* and *S* labels refer to the absolute configuration of C(1) in each indenyl ring, as shown in Scheme 4. 

**Figure 3:**
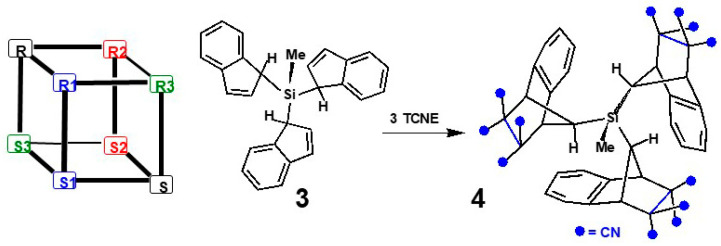
(**Left**) The cube representing exchange between eight different indenyl environments in **3**, and (**right**) the product of triple Diels-Alder addition of TCNE to tris(indenyl)methylsilane, **4**.

A combination of ^1^H-^1^H COSY, ^1^H-^13^C and ^1^H-^29^Si shift-correlated NMR data revealed the unequivocal assignment of the proton and carbon-13 nuclei in all the different indenyl ring environments, and 1D-selective inversion and 2D-EXSY spectra allowed the elucidation of their molecular dynamics [17]. As depicted in Figure 3, the exchange pathways between indenyl sites in **3** can be mapped onto a cube (for the eight different indenyl ring environments), and a hypercube (for the exchange of ^1^H in sp^2^ and sp^3^ environments) and again involve successive [1,5]-suprafacial sigmatropic shifts via isoindene intermediates. Gratifyingly, several triple Diels-Alder TCNE adducts, **4**, have been isolated and fully characterized by X-ray crystallography [18,19].

**Figure 4:**
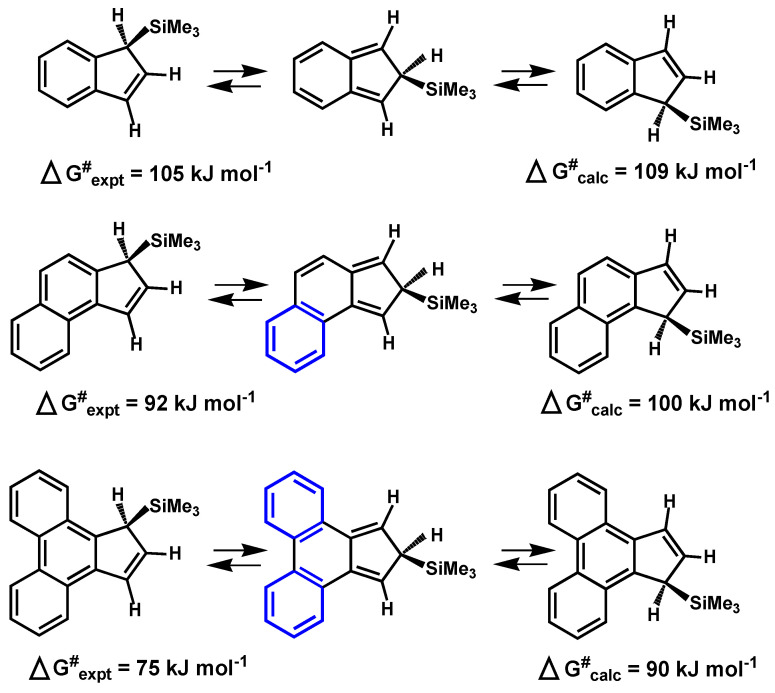
Calculated and experimental barriers to silatropic shifts in a series of benzindenes.

Comparison of silyl migrations across a series of benzo- and dibenzo-indenes revealed a diminution of the barrier whereby the additional benzo rings enhanced the aromatic character of the intermediate isoindenes. Typically, calculations at the unrestricted Hartree-Fock level yielded a barrier of 109 kJ mol^−1^ for trimethylsilylindene, 100 kJ mol^−1^ for the angular benzindene and 90 kJ mol^−1^ for the dibenzindene (trimethylsilylcyclopenta[*l*]phenanthrene). As before, all the isoindenes underwent ready Diels-Alder addition with TCNE. Evidently, additional benzo rings stabilise the isoindene. Indeed, in the dibenzindene the intermediate lies only 9 kJ mol^−1^ above the ground state. Gratifyingly, the experimentally determined migration barriers correlate well with the theoretical predictions [20,21,22], as listed in Figure 4.

**Figure 5:**
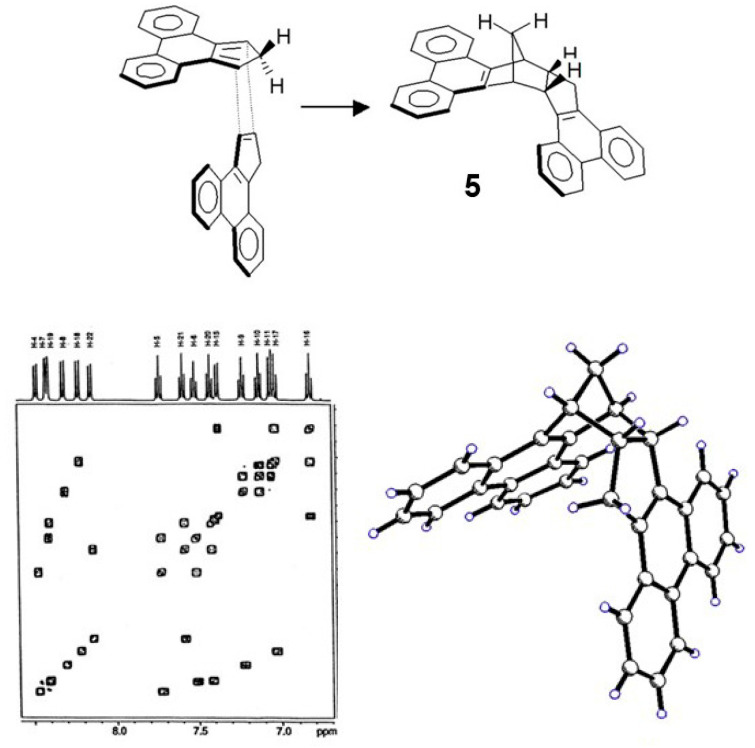
500 MHz ^1^H-^1^H COSY NMR spectrum in the aromatic region, and molecular structure, of the Diels-Alder dimer formed by reaction of cyclopenta[*l*]phenanthrene with its own isoindene [23]. Figure 5 is reprinted with permission of the American Chemical Society.

This latter result provides an ideal rationale for the observation that, when cyclopenta[*l*]phenanthrene was heated at reflux in dibutyl ether, the product (85% yield) was the dimer, **5**, shown in Figure 5 [23]. Its structure was established initially via a beautifully resolved ^1^H-^1^H-COSY NMR spectrum, together with other 2D NMR data, and subsequently by X-ray crystallography as the Diels-Alder adduct of the starting material with its own isoindene—a striking demonstration of the stabilising effect of the additional benzo rings!

## 3. Diels-Alder Cycloadditions of Alkynes or Triphenylcyclopropene to Cyclopentadienones

### 3.1. Geometric Factors in C_n_Ph_n_ Ring Systems

In the solid state, ligands or complexes possessing a cyclic array of C_n_Ph_n_ moieties adopt propeller-type arrangements such that the external rings make a dihedral angle, *θ*, with the plane of the central ring. Such a geometry provides a compromise between the coplanar arrangement, *θ* = 0°, which maximises orbital overlap but may introduce steric strain, and the orthogonal rotamer, *θ* = 90°, that minimises steric interactions but disrupts π conjugation. Furthermore, in the series C_n_Ph_n_, where n = 3–7, the angle subtended at the centre of the internal ring by the adjacent phenyls (ω = 360°/n) decreases from 120°, 90°, 72°, 60° to 51.4°, respectively. However, although increasing the ring size lengthens the radial distance of the external phenyls from the centre of the molecule, this is more than compensated for by the diminishing value of ω. Overall, the net result of increasing the ring size is to place the phenyls in a more restricted locale [24].

**Scheme 5:**
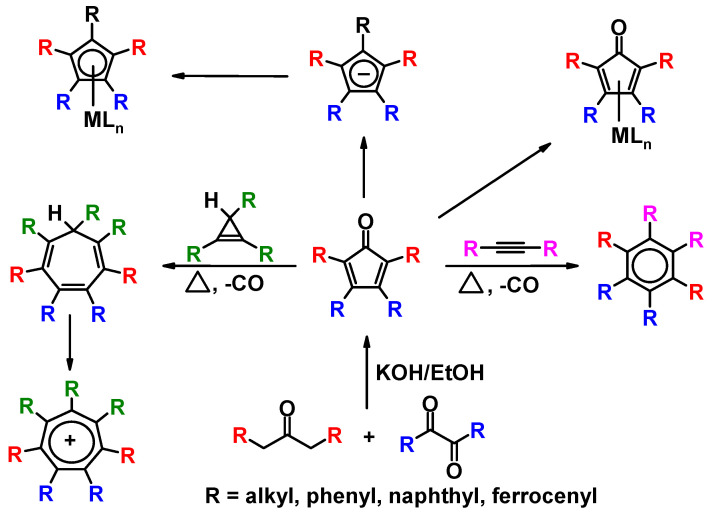
Cyclopentadienones as precursors to five, six and seven-membered ring systems.

Cyclopentadienones are versatile precursors to organic and organometallic derivatives of five, six and seven-membered rings, as exemplified in Scheme 5. Typically, reactions of tetraarylcyclopentadienones and alkynes provide convenient routes to multifunctionalised arenes and their organometallic derivatives, in particular those bearing several sterically demanding substituents. The syntheses and molecular dynamics of such systems have been reviewed [24]. 

Ions or molecules of the type [C_n_Ar_n_]^x±^ continue to attract attention, not only for their relevance to the Hückel 4n + 2 rule, but also as ligands bonded to organometallic fragments. Thus, [C_3_Ph_3_^+^], [C_4_Ph_4_^2^^−^], [C_5_Ph_5_^−^], C_6_Ph_6_ and [C_7_Ph_7_^+^] would be expected to exist as stable species. Indeed, those where n ranges from 2 to 6, have all been characterised crystallographically as free ligands or as metal complexes. However, the structure of the [C_7_Ph_7_^+^] motif remained a challenge for almost four decades after its initial preparation.

### 3.2. Syntheses and Structures of Heptaarylcycloheptatrienes

The first synthesis of heptaphenylcycloheptatriene, **6**, was reported by Battiste in 1961 from the Diels-Alder cycloaddition of 1,2,3-triphenylcyclopropene to tetracyclone (tetraphenyl-cyclopentadienone) in refluxing xylene [25,26]. Reaction with bromine in CCl_4_ furnished the bright red salt [C_7_Ph_7_^+^]Br^−^. The yellow-orange tribromide, [Br_3_^−^] and tetrafluoroborate salts were also prepared. Subsequently, the corresponding heptaphenylcycloheptatrienyl anion, [C_7_Ph_7_^−^] and the radical [C_7_Ph_7_^•^] were characterised spectroscopically [27,28]. In the former case, the 8π electron count might have suggested a triplet ground state (analogous to 4π cyclobutadiene), but the observation of a well-defined ^1^H NMR spectrum and failure to detect an EPR signal indicated a singlet structure, implying a lowering of the seven-fold symmetry.

**Figure 6:**
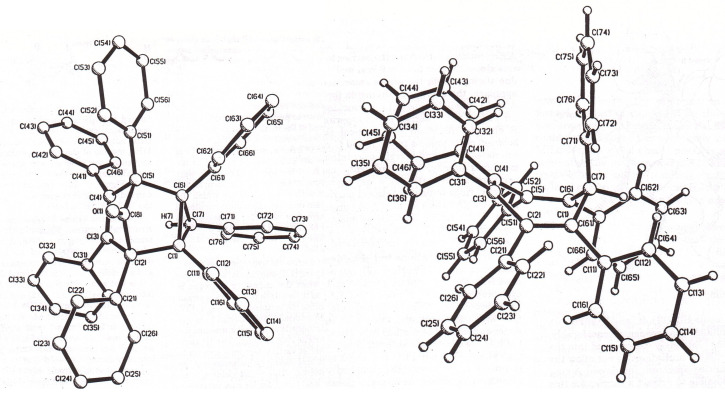
Molecular structures of (**left**) the Diels-Alder adduct of tetracyclone and triphenylcyclopropene, **7**, and (**right**) heptaphenylcycloheptatriene, **6** [29]. Figure 6 is reprinted with permission of the American Chemical Society.

Heptaphenylcycloheptatriene prepared by the Battiste method, was formed directly with elimination of carbon monoxide when triphenylcyclopropene and tetracyclone were heated in refluxing xylene. However, when these reagents were stirred at room temperature for six days, the intermediate ketone, **7**, was obtained in 80% yield [29]. As shown in Figure 6, all three cyclopropyl phenyls in **7** are exo and, most importantly, the single hydrogen is positioned endo [29]. This is in accord with Battiste’s report that, in the Diels-Alder adduct of triphenylcyclopropene and cyclopentadiene, the single hydrogen is also endo, but that assignment was based only on NMR data [30].

The structure of heptaphenylcycloheptatriene also appears in Figure 6 and reveals that the unique phenyl substituent is axial and straddles the molecular mirror plane. The molecule adopts a boat conformation such that the fold angles between planes containing C(6)-C(7)-C(1) [plane 1], C(1)-C(2)-C(5)-C(6) [plane 2] and C(2)-C(3)-C(4)-C(5) [plane 3] are 55° for [plane 1/plane 2] and 35° for [plane 2/plane 3]. These may be compared with the corresponding interplanar angles in C_7_H_8_, which are 36° for [plane 1/plane 2] and 40° for [plane 2/plane 3]. Thus, the conformation of the seven-membered boat in C_7_Ph_7_H is markedly different from that found in cycloheptatriene itself [31], especially with respect to the greater bending of the sp^3^ carbon out of the C(1)-C(2)-C(5)-C(6) plane (55° v 36°). 

**Figure 7:**
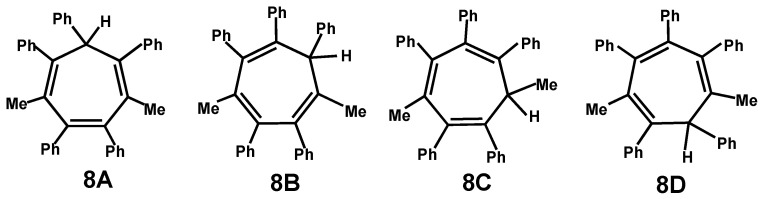
The four possible isomers of **8**, arising from [1,5]-hydrogen shifts upon ring-opening of the Diels-Alder adduct of triphenylcyclopropene and 2,5-dimethyl-3,4-diphenylcyclopentadienone.

The corresponding cycloaddition reaction of triphenylcyclopropene and 2,5-dimethyl-3,4-diphenylcyclopentadienone (which occurs as its Diels-Alder dimer, and must therefore be cracked before use) yielded the expected cycloheptatriene C_7_Ph_5_Me_2_H, **8**. However, the complexity of the ^1^H and ^13^C NMR spectra, in particular the nonequivalence of the methyl groups in both regimes, revealed that the product cannot be the anticipated symmetrical isomer **8A**, shown in Figure 7. Moreover, the singlet character of both methyl signals in the ^1^H spectrum eliminates **8C** and leaves **8B** and **8D** as the only viable candidates. The identification of the product as **8B** was determined by a combination of 2D and nOe experiments [29]. 

**Figure 8:**
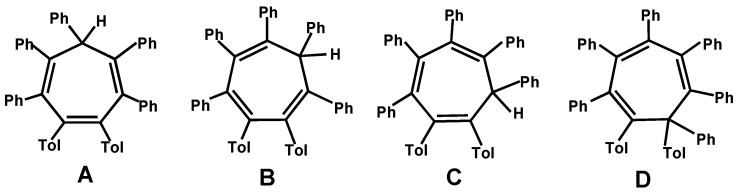
The four isomers (**A**–**D**) arising from [1,5]-hydrogen shifts upon ring-opening of the Diels-Alder adduct of triphenylcyclopropene and 2,5-diphenyl-3,4-di-(*p*-tolyl)cyclopentadienone.

Similarly, the reaction of triphenylcyclopropene and 2,5-diphenyl-3,4-di-(*p*-tolyl)-cyclopentadienone proceeded with elimination of CO and furnished a mixture giving rise to seven equally intense ^1^H NMR methyl resonances, attributable to the four isomers A through D in the ratio 1:2:2:2, as shown in Figure 8. 

**Scheme 6:**
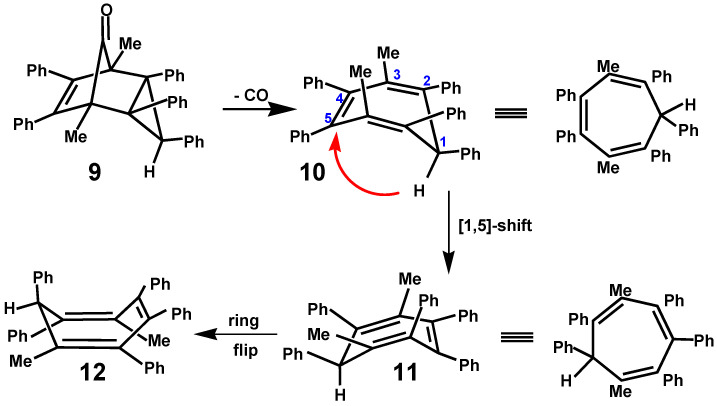
[1,5]-Hydrogen shifts in hepta-arylcycloheptatrienes.

Evidently, such molecules must arise via hydrogen migrations, but the symmetry-allowed [1,5]-suprafacial sigmatropic shift can only occur when the migrating hydrogen is positioned axially to facilitate the rearrangement shown in Scheme 6. Apparently, after cheletropic elimination of CO and opening of the three-membered ring in **9**, the conformation of the newly generated heptaphenylcycloheptatriene, **10**, must be sufficiently long-lived to allow rapid [1,5] suprafacial sigmatropic shifts, as in **11**, before ring flipping to the other boat conformation, **12**, prevents any further hydrogen migrations [29].

### 3.3. The Heptaphenyltropylium Cation

As noted above, in 1961 Battiste prepared salts of the heptaphenyltropylium cation with bromide, tribromide and tetrafluoroborate counterions [26]. With the aim of extending the C_n_Ph_n_ series to include [C_7_Ph_7_^+^], we acquired numerous X-ray data sets on crystals of the bromide and tetrafluoroborate salts but, despite the determined efforts of a number of crystallographers in Europe and North America, no structural data were forthcoming because of an unresolvable disorder problem. Serendipitously, however, in an NMR tube containing C_7_Ph_7_Br in trifluoroacetic acid, formation of crystalline red plates was observed, and the resulting data set was resolvable. In fact, the crystals were found to be [C_7_Ph_7_^+^][CF_3_CO_2_^−^](CF_3_CO_2_H)_2_ in which the cations were arranged in hexagonally packed layers that sandwiched a network of hydrogen-bonded trifluoracetates and trifluoroacetic acid molecules [32]. Evidently, this acid-anion network stabilises the crystalline edifice, whereas in the earlier cases the bromides or tetrafluoroborates apparently behaved merely as layers of molecular ball-bearings that lacked directionality and failed to impose order on the system. 

**Figure 9:**
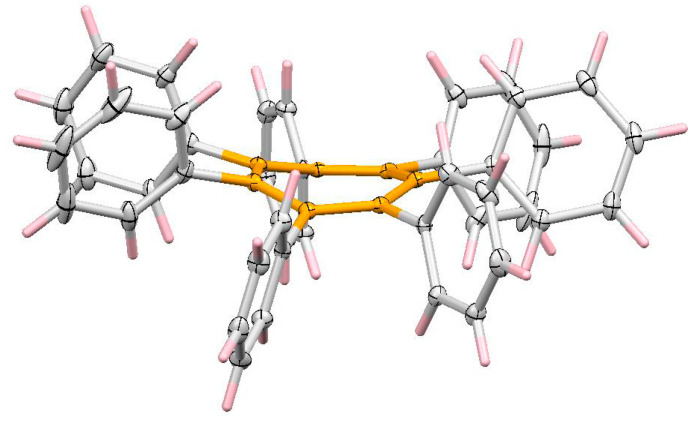
Molecular structure of the nonplanar heptaphenyltropylium cation, C_7_Ph_7_^+^, **13**.

**Figure 10:**
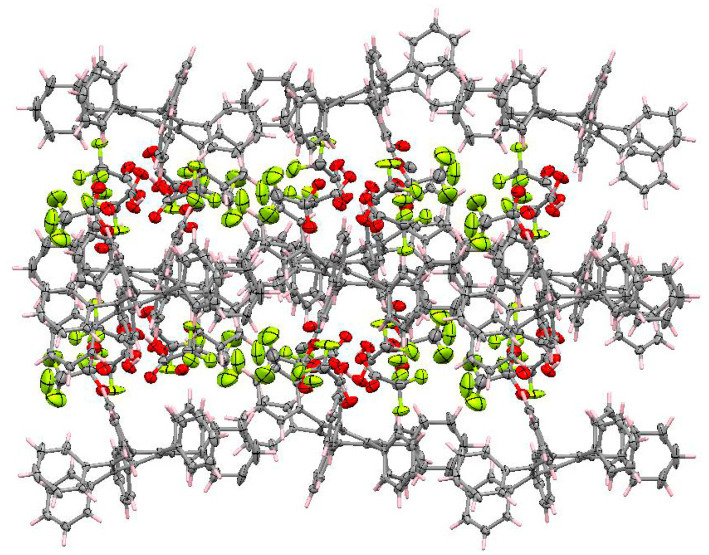
View of the crystal packing of [C_7_Ph_7_^+^][CF_3_CO_2_^−^](CF_3_CO_2_H)_2_ depicting the alternating layers of cations (grey) and anions/solvates (red/yellow).

The molecular structure of the cation, **13**, and of the crystal packing, are shown as Figure 9 and Figure 10 and reveal that the seven-membered ring is not planar but adopts a shallow boat conformation such that the interplanar angles between C(6)-C(7)-C(1) [plane 1], C(1)-C(2)-C(5)-C(6) [plane 2], and C(2)-C(3)-C(4)-C(5) [plane 3] are 13° for [plane 1/plane 2] and 18° for [plane 2/plane 3], noticeably less bent than those found for the precursor C_7_Ph_7_H. The peripheral phenyls are each twisted very markedly out of the plane containing their attached central ring carbon and neighbouring ring atoms by 76°–82° (average 80°), and may be compared to the values for dihedral angles found in C_6_Ph_6_ (67°–75°) [33,34], (η^5^-C_5_Ph_5_)ML_n_ complexes (~50°) [35,36,37], (η^4^-C_4_Ph_4_)ML_n_ complexes (average ~36°) [38,39] and for the [C_3_Ph_3_^+^] cation (~5°) [40,41].

### 3.4. Boron and Nitrogen Analogues of Heptaphenylcycloheptatrienes

**Scheme 7:**
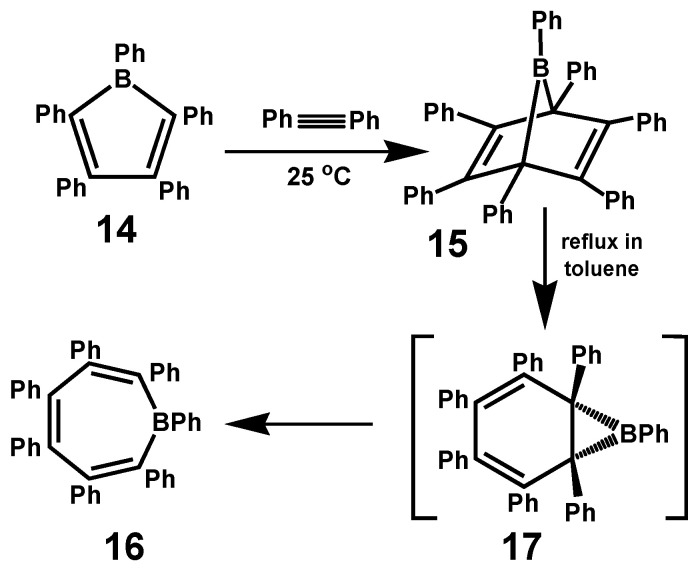
Synthetic route to heptaphenylborepin, C_6_BPh_7_.

In a closely analogous system, addition of diphenylacetylene to pentaphenylborole, **14**, yielded heptaphenyl-7-borabicyclo[2.2.1]heptadiene, **15**, which, upon heating at reflux in toluene for 24 h, formed heptaphenylborepin, **16** [42]. One can envisage this proceeding via a migration of the boron to form the [4.1.0]bicyclo isomer, **17**, that undergoes disrotatory ring opening to furnish the target molecule (Scheme 7). The relationship with the isoelectronic heptaphenyltropylium ion is reflected in its electronic spectrum which exhibits peaks at 412, 276 and 245 nm (405, 283 and 258 nm in C_7_Ph_7_^+^). It would be interesting to know whether heptaphenylborepin is planar or nonplanar. However, we are unaware of any relevant X-ray structural data.

**Scheme 8:**
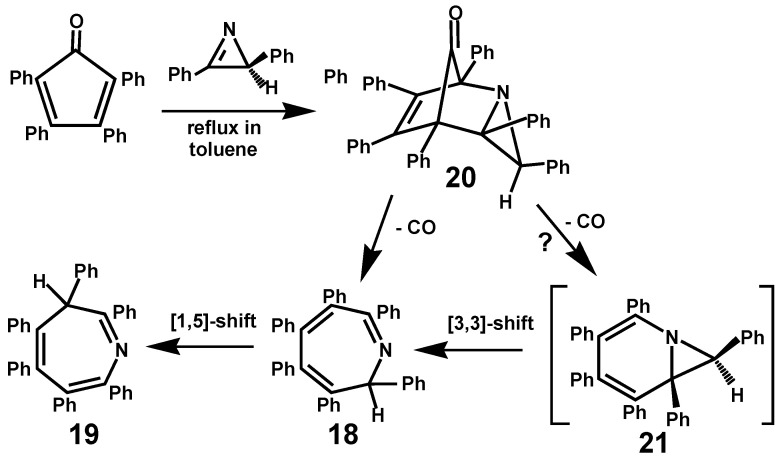
Synthetic route to hexaphenylazepin, C_6_NPh_7_H.

By way of contrast, the reaction of tetracyclone with diphenylazirine yields initially the 2*H*-hexaphenylazepine, **18**, that subsequently undergoes a [1,5]-hydrogen shift to form the thermodynamically favoured 3*H*-hexaphenylazepine, **19**. The proposed mechanism (Scheme 8) invokes Diels-Alder addition to form the intermediate **20**, in which the hydrogen is positioned endo. Loss of CO could then either bring about ring opening directly to form 2*H*-hexaphenylazepine, or proceed via the azanorcaradiene, **21**, that undergoes disrotatory electrocyclic rearrangement. Formation of another possible isomer, the 1*H*-azepine was not observed. In some related cases, the 1-azirine was prepared in situ by thermolysis of the appropriate vinyl azide [43].

### 3.5. Ferrocenyl-Substituted Polyaryl-Benzenes and -Cycloheptatrienes

**Scheme 9:**
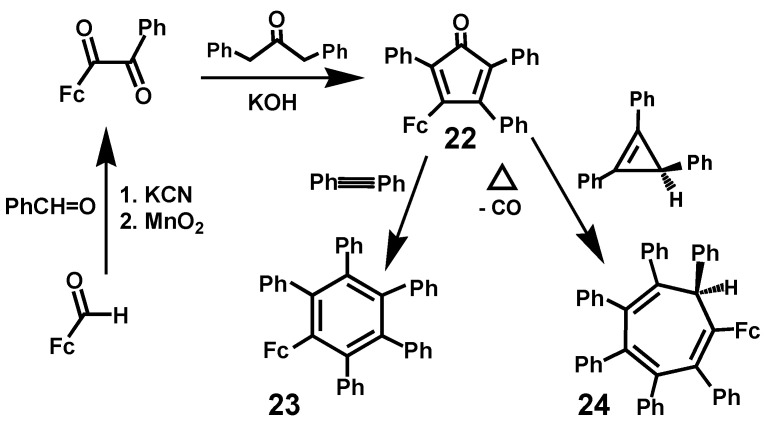
Synthetic routes to C_6_Ph_5_Fc, **23**, and C_7_Ph_6_FcH, **24**.

**Figure 11:**
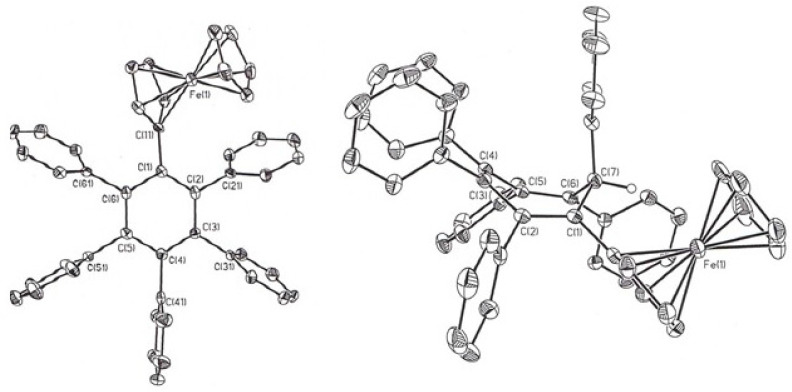
Molecular structures of (**left**) ferrocenylpentaphenylbenzene, **23**, and (**right**) ferrocenylhexaphenylcycloheptatriene, **24**. Figure 11 is reprinted with permission of the American Chemical Society.

The successful elucidation of the structure of the heptaphenyltropylium ion prompted us to consider the introduction of a ferrocenyl substituent that might enhance the stability of the potential cation [C_7_Ph_6_Fc^+^], where Fc = (η^5^-C_5_H_4_)Fe(η^5^-C_5_H_5_). To this end, the KCN-catalysed reaction of benzaldehyde and formylferrocene yielded the benzoin PhC(=O)-CH(OH)Fc. It is noteworthy that only a single benzoin was formed; the other possible isomer, FcC(=O)-CH(OH)Ph, would require the intermediacy of a carbanion adjacent to the ferrocenyl fragment which, as is well known, preferentially stabilises cations [44]. Oxidation of the benzoin to form the corresponding benzil, and reaction with dibenzyl ketone, furnished the required 3-ferrocenyl-2,4,5-triphenyl-cyclopentadienone, **22**. This versatile precursor reacts with diphenylacetylene to form ferrocenylpentaphenylbenzene, **23**, and also with triphenylcyclopropene to generate ferrocenylhexaphenylcycloheptatriene, **24**, (Scheme 9), both of which have been characterised by X-ray crystallography (Figure 11) [45].

**Figure 12:**
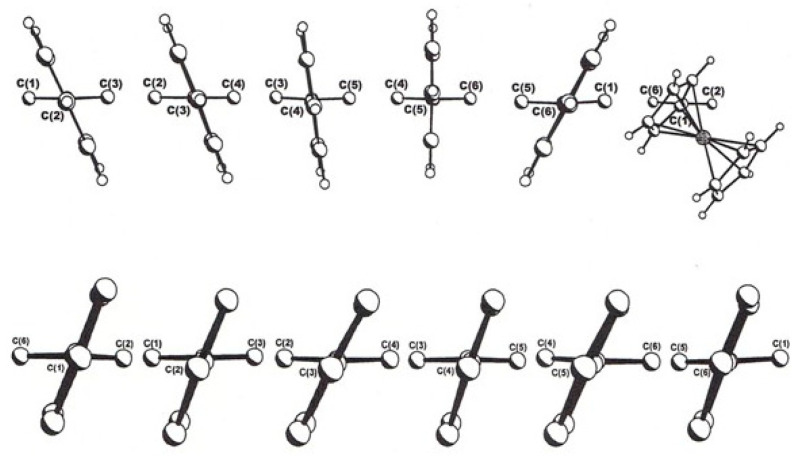
View along successive peripheral-ring–central-ring axes showing the sequence of peripheral phenyl torsion angles (top) in C_6_Ph_5_Fc and (bottom) in C_6_Ph_6_.

A curious feature of the structure of ferrocenylpentaphenylbenzene is the orientation of the peripheral phenyls relative to the central ring. In hexaphenylbenzene, the molecule adopts a propeller conformation in which the phenyl substituents are all canted in the same direction with a twist angle of ca. 67° [33]. However, in C_6_Ph_5_Fc the ferrocenyl ring bonded to C(1) is oriented at 51° to the central ring, and the phenyls attached to C(2) through C(6) adopt dihedral angles of 64°, 70°, 81°, 89° and 120° (Figure 12). The net effect is to provide a series of peripheral rings each displaced slightly more than their immediately preceding neighbours. One is tempted to suggest that rotation of the ferrocenyl moiety would induce a domino effect whereby all the phenyls would turn in a synchronous fashion, but verification of such a scenario would require extensive labelling studies. 

Turning now to the structure of ferrocenylhexaphenylcycloheptatriene, **24**, the seven-membered ring adopts a boat conformation such that the fold angles between planes containing C(6)-C(7)-C(1) [plane 1], C(1)-C(2)-C(5)-C(6) [plane 2], and C(2)-C(3)-C(4)-C(5) [plane 3] are 54° for [plane 1/plane 2] and 34° for [plane 2/plane 3], similar to those seen in C_7_Ph_7_H. Once again, the peripheral substituents in C_7_Ph_6_FcH are markedly twisted out of the plane defined by their attached central ring carbon and its neighbours. These dihedral angles were found to be 56°, 82°, 81°, 68°, 55°, 132° and 142° for positions C(1) through C(7) [45]. At this point, we recall that in suitably labelled polyphenylcycloheptatrienes a series of [1,5]-hydrogen shifts generated a mixture of isomers [29]. However, despite the complexity of the ^1^H and ^13^C NMR spectra of **24**, it transpired that only a single isomer was formed from the Diels-Alder cycloaddition of triphenylcyclopropene and 3-ferrocenyl-2,4,5-triphenylcyclopentadienone. The X-ray crystal structure revealed that the ferrocenyl substituent is sited in the least hindered position, at C(1) adjacent to the sp^3^-hybrised carbon of the CHPh unit. Clearly, this isomer must have arisen by a [1,5]-hydrogen migration after decarbonylation, but prior to inversion of the cycloheptatriene ring, as illustrated for the C_7_Ph_5_Me_2_H system in Scheme 6. 

**Scheme 10:**
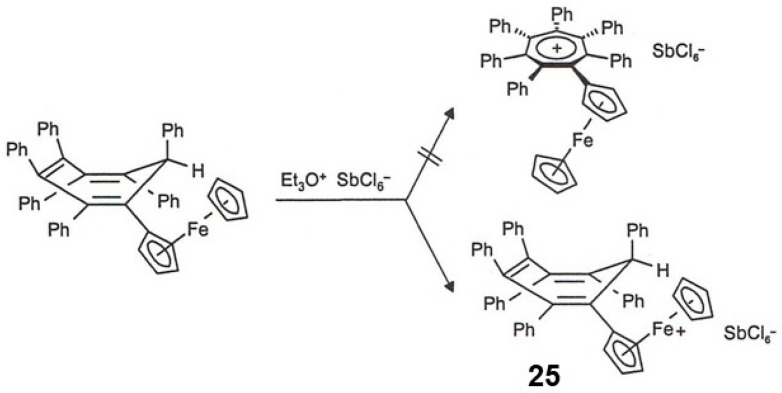
Attempted preparation of the [C_7_Ph_6_Fc^+^] cation leads instead to oxidation of the iron.

As noted above, one aim of the preparation of **23** was to attempt the isolation of the potential cation [C_7_Ph_6_Fc^+^]. To this end, we tried to isolate the molecules in which the single hydrogen in C_7_Ph_6_FcH had been replaced with bromide or methoxide, but to no avail. Finally, treatment of **23** with triethyloxonium hexachloroantimonate, a known hydride abstractor, yielded deep blue crystals that were submitted for X-ray crystallographic analysis. Disappointingly, this revealed the product to be [C_7_Ph_6_FcH^+^][SbCl_6_^−^], **25**, the ferricenium salt of the starting material (Scheme 10). The structure of the cation closely resembles that of C_7_Ph_6_FcH, with only minor changes in the ferrocenyl unit, and of course the presence of the hexachloroantimonate counterion. It may be the case that the steric bulk of the ferrocenyl unit hinders the approach of the triethyloxonium reagent and, instead, an electron transfer process intervenes [45].

### 3.6. Hexa(β-naphthyl)benzene and Ferrocenyl-penta(β-naphthyl)benzene

**Figure 13:**
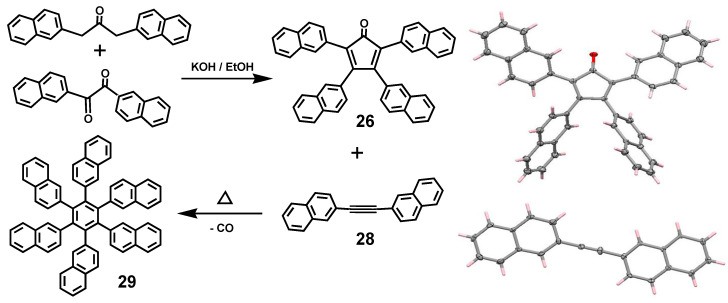
(**left**) synthetic route to hexa(β-naphthyl)benzene, and (**right**) molecular structures of tetra(β-naphthyl)cyclopentadienone, **26**, and di(β-naphthyl)acetylene, **28**.

The analogous naphthyl-substituted systems, tetra(β-naphthyl)cyclopentadienone, **26**, and 3-ferrocenyl-2,4,5-tri(β-naphthyl)cyclopentadienone, **27**, have also been prepared and their Diels-Alder reactivity exploited [46,47]. As shown in Figure 13, tetra(β-naphthyl)cyclopentadienone and di(β-naphthyl)acetylene, **28**, undergo [4 + 2] cycloaddition and, after decarbonylation, yield hexa(β-naphthyl)benzene, **29**.

**Scheme 11:**
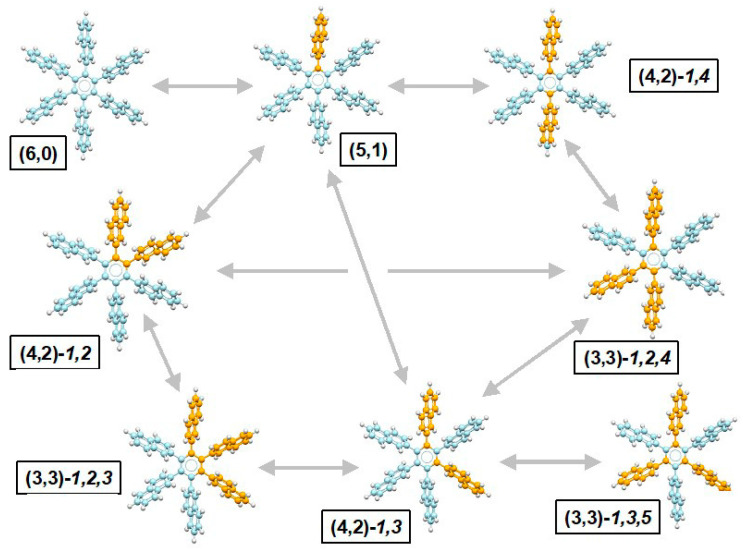
The eight energy-minimised rotamers of hexa(β-naphthyl)benzene, **29**, showing which can be directly interconverted by rotation of a single peripheral ring.

In hexa(β-naphthyl)benzene the six peripheral substituents are oriented in a propeller fashion with dihedral angles of 60° [46]. However, there is a disorder because the molecule exists as a mixture of eight conformers whereby the naphthyls can be aligned all syn, i.e., 6:0, or in a 5:1 arrangement, or 4:2 (1,2), (1,3) or (1,4), or 3:3 (1,2,3), (1,2,4) or (1,3,5). Assuming independent rotation of one peripheral ring at a time, these can interconvert according to Scheme 11 [46].

**Figure 14:**
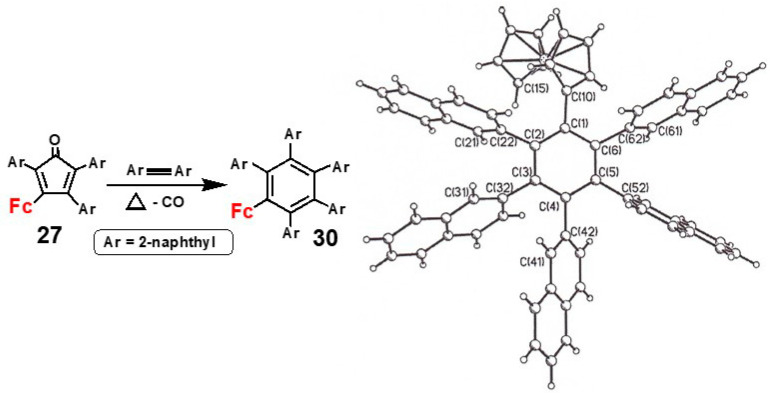
Synthesis and molecular structure of ferrocenyl-penta(β-naphthyl)benzene, **30**. Figure 14 is reprinted from the Canadian Journal of Chemistry; © Canadian Science Publishing or its licensors.

In contrast, in ferrocenyl-penta(β-naphthyl)benzene, **30**, (Figure 14), the orientations of the peripheral β-naphthyl rings paralleled the behaviour of the phenyls in C_6_Ph_5_Fc whereby each ring was rotated more than its preceding neighbour. The dihedral angles of ferrocenyl at C(1) and naphthyls attached to C(6) through C(2) adopt dihedral angles of 134°, 106°, 81°, 62°, 65° and 73°, respectively [47]. 

### 3.7. Other Organometallic Derivatives of C_6_Ph_6_ and C_7_Ph_7_H

**Scheme 12:**
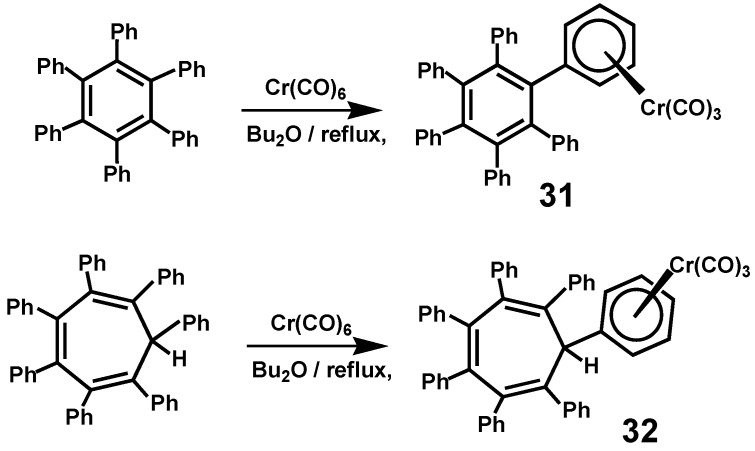
Reactions of C_6_Ph_6_ and C_7_Ph_7_H with chromium hexacarbonyl.

Having successfully prepared the ferrocenyl derivatives **23**, **24** and **30**, routes to other organometallic complexes of these sterically hindered systems were investigated. The reaction of hexaphenylbenzene with chromium hexacarbonyl leads to formation of the complex (C_6_H_5_)_5_C_6_[C_6_H_5_(η^6^-Cr(CO)_3_)], **31**, in which the metal is coordinated to a peripheral ring [48]. Likewise, as illustrated in Scheme 12, the analogous reaction with C_7_Ph_7_H yields (C_6_H_5_)_6_C_7_H[C_6_H_5_(η^6^-Cr(CO)_3_], **32**, in which the chromium is attached to the unique phenyl in the axial position [29]. This contrasts with the situation in 7-phenylcycloheptatriene in which the metal tripod is η^6^-bonded to the 7-membered ring [49]. 

**Figure 15:**
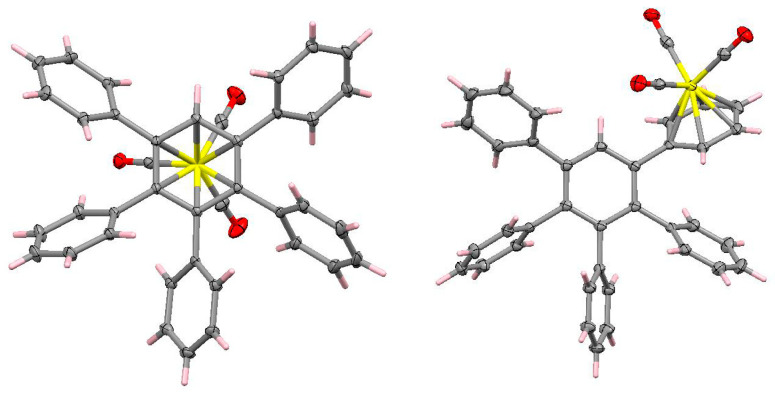
Molecular structures of (C_6_Ph_5_H)Cr(CO)_3_; (**left**) tripod bonded centrally, **33**; (**right**) tripod bonded to a peripheral ring in **34**.

In the somewhat less sterically hindered system, pentaphenylbenzene (the Diels-Alder adduct of tetracyclone and phenylacetylene) the reaction with chromium hexacarbonyl yielded two π-bonded Cr(CO)_3_ derivatives (Figure 15): the centrally bonded isomer, **33**, and the peripherally complexed molecule, **34**, in which the metal tripod is bonded to the least hindered phenyl adjacent to the ring hydrogen [50].

**Scheme 13:**
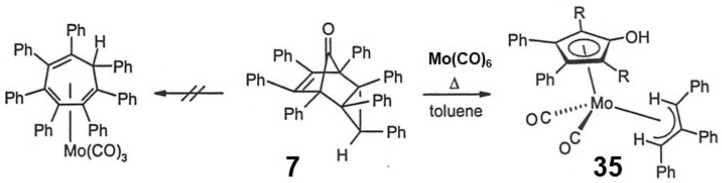
Reaction of the ketone **7** with molybdenum hexacarbonyl.

**Scheme 14:**
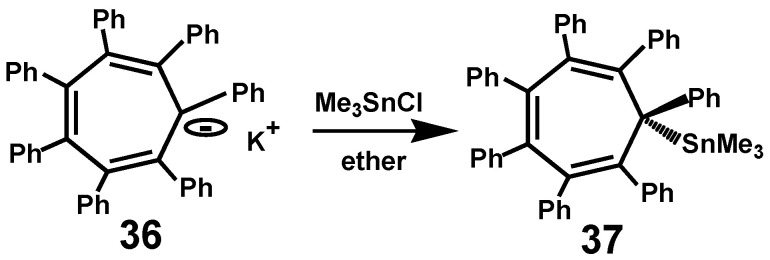
Reaction of C_7_Ph_7_K with chlorotrimethylstannane.

Attempts to coordinate molybdenum or tungsten tricarbonyls to the central ring of C_7_Ph_7_H were unsuccessful and so a different potential route, involving reaction with the primary Diels-Alder ketone adduct, **7**, was considered. The intent was to try to coordinate a molybdenum carbonyl fragment onto its the open face in the expectation that subsequent metal-assisted cyclopropane ring opening and loss of CO would yield a complex in which the metal was bonded to the central ring. However, the isolated product was instead characterised by X-ray crystallography as the complex, **35**, in which a Mo(CO)_2_ moiety was η^5^-bonded to a 1-hydroxy-2,3,4,5-tetraphenylcyclopentadienyl ligand and also η^3^-linked to a triphenyl allyl fragment (Scheme 13). Apparently, the ketone underwent a retro Diels-Alder process and suffered opening of the 3-membered ring [29]. Greater success was achieved by treatment of C_7_Ph_7_Br with potassium metal to form the deep blue anion [C_7_Ph_7_^−^], **36**, that reacted with chlorotrimethylstannane or chlorodimethylsilane to form yellow σ-bonded complexes, as in **37** (Scheme 14).

**Scheme 15:**
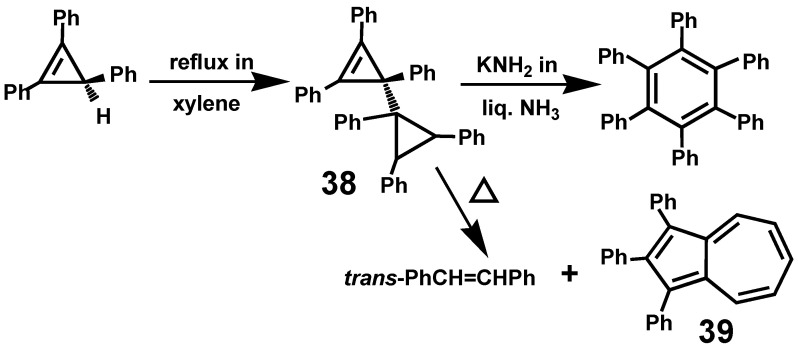
Reactivity of triphenylcyclopropene.

We note in passing that, although triphenylcyclopropene can be used in Diels-Alder reactions, one should recall that when heated at reflux in xylene it dimerises to yield 3-triphenylcyclopropyl-triphenylcyclopropene, **38**. Subsequent treatment with KNH_2_ furnishes hexaphenylbenzene, whereas continued thermolysis leads to the ring expansion product triphenylazulene, **39**, with elimination of trans-stilbene as shown in Scheme 15 [51].

### 3.8. Organometallic Derivatives of Persubstituted Cycloheptatrienes 

Other per-substituted seven-membered ring systems include C_7_Cl_8_ [52], C_7_(CO_2_Me)_7_H [53], C_7_Me_7_H [54] and the aforementioned heptaphenylborepin BC_6_Ph_7_ [42]. Furthermore, a number of bis(heptaphenylcycloheptatrienes) have been prepared by double Diels-Alder addition of triphenylcyclopropene to a series of linked bis(tetracyclone) precursors [55]. 

#### 3.8.1. Octachlorocycloheptatriene, C_7_Cl_8_

**Scheme 16:**
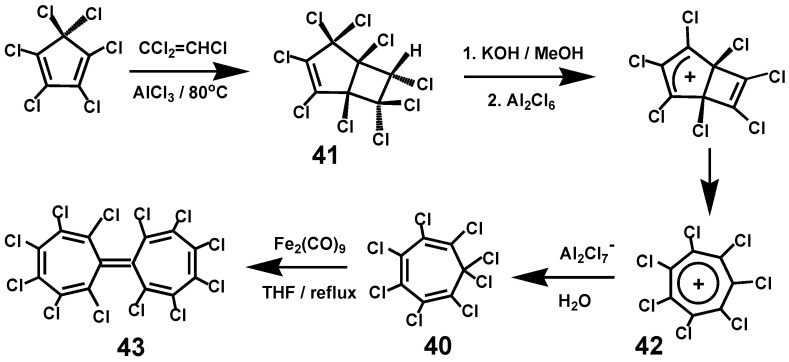
Synthesis of octachlorocycloheptatriene.

**Figure 16:**
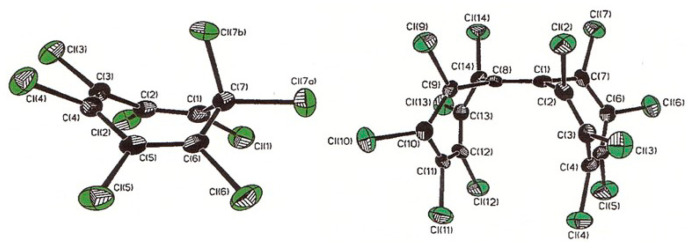
Structures of (**left**) octachlorocycloheptatriene, **40**, and (**right**) dodecachlorofulvalene, **43**. Figure 16 is reprinted from the Canadian Journal of Chemistry; © Canadian Science Publishing or its licensors.

Perchlorocycloheptatriene, **40**, was originally prepared by West and coworkers by cycloaddition of hexachlorocyclopentadiene with trichloroethylene [52]. However, this proceeds via a [2 + 2] rather than a [4 + 2] cycloaddition to form the bicyclo[3.2.0]heptane, **41**, which, after elimination of HCl and reaction with AlCl_3_ at 150 °C, forms the perchlorotropylium ion, **42**, that recaptures a chloride from the heptachlorodialuminate anion (Scheme 16). The boat conformation of C_7_Cl_8_ has been determined X-ray crystallographically (Figure 16) and revealed that the fold angles between planes containing C(6)-C(7)-C(1) [plane 1], C(1)-C(2)-C(5)-C(6) [plane 2], and C(2)-C(3)-C(4)-C(5) [plane 3] are 52° for [plane 1/plane 2] and 32° for [plane 2/plane 3], similar to those found in C_7_Ph_7_H. Reaction with a number of metal carbonyls led to formation of the dodecachlorofulvalene, **43**, possibly via a metal carbene intermediate [56].

#### 3.8.2. The heptamethyltropylium Cation, [C_7_Me_7_^+^]

**Scheme 17:**
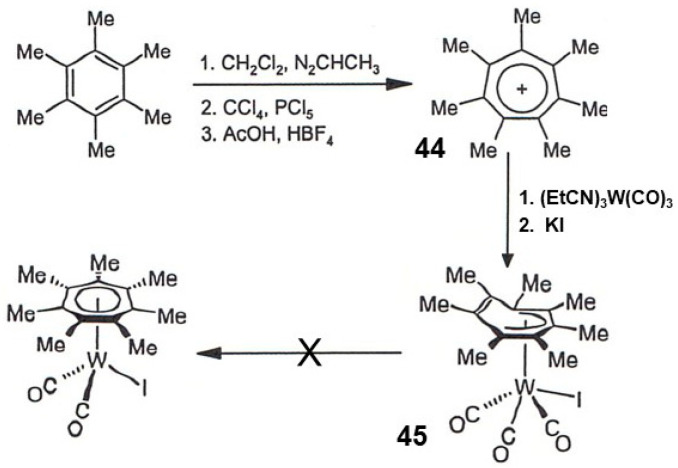
[C_7_Me_7_^+^] reacts to form the complex (η^5^-C_7_Me_7_)W(CO)_3_I, but not (η^7^-C_7_Me_7_)W(CO)_2_I.

We note that in a comprehensive study by Tamm, heptamethylcycloheptatriene C_7_Me_7_H, formed via ring expansion of hexamethylbenzene by a carbene, rather than by a Diels-Alder reaction, and also its corresponding cation [C_7_Me_7_^+^], **44**, have been prepared and fully characterised [54]. The cation was shown to adopt a nonplanar boat conformation such that the fold angles between planes containing C(6)-C(7)-C(1) [plane 1], C(1)-C(2)-C(5)-C(6) [plane 2], and C(2)-C(3)-C(4)-C(5) [plane 3] were 21° for [plane 1/plane 2] and 30° for [plane 2/plane 3], noticeably larger than those seen in the heptaphenyltropylium cation. This may be rationalised in terms of the greater steric demand of the three-dimensional methyl groups relative to the flat phenyl substituents that can rotate markedly out of the central plane. Reaction of the cation with (EtCN)_3_W(CO)_3_ and sodium iodide was expected to yield (η^7^-C_7_Me_7_)W(CO)_3_I, but in fact the product isolated was (η^5^-C_7_Me_7_)W(CO)_3_I, **45**, in which the uncoordinated double bond is folded away from the complexed 5-atom plane through 60°, as shown in Scheme 17, once again emphasising the severe steric hindrance engendered by the seven methyl substituents [57].

#### 3.8.3. Heptacarbomethoxycycloheptatriene, C_7_(CO_2_Me)_7_H

**Scheme 18:**
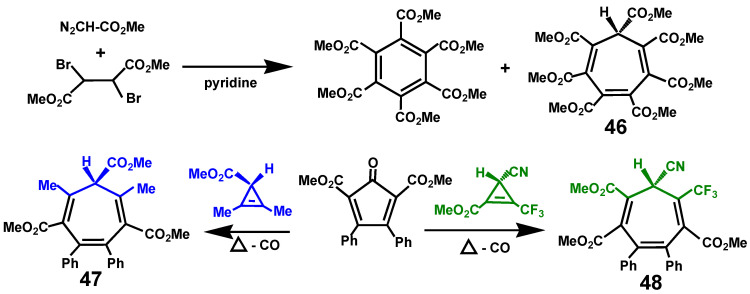
Diels-Alder reactions of cyclopentadienones and cyclopropenes bearing multiple electron-withdrawing substituents.

In more recent work, Tomilov and coworkers reported that the cascade reaction of methyl diazoacetate and dimethyl dibromosuccinnate in the presence of pyridine furnished the hexa- and hepta-esters, C_6_(CO_2_Me)_6_ and C_7_(CO_2_Me)_7_H (Scheme 18). The cycloheptatriene and its potassium salt were both characterised by X-ray diffraction. The boat conformation in C_7_(CO_2_Me)_7_H, **46**, parallels that seen previously seen with fold angles of 54° for [plane 1/plane 2] and 33° for [plane 2/plane 3], compared to 55° and 35° in C_7_Ph_7_H, 52° and 32° in C_7_Cl_8_. However, in the potassium salt, the seven-membered anionic ring flattens such that these angles change dramatically to become 29° and 43°. This is indicative of partial conjugation involving five ring carbons with delocalisation of the negative charge onto the ester oxygens [53].

Although the hepta-ester was not preparable directly via a Diels-Alder route, reaction of 2,5-dicarbomethoxy-3,4-diphenylcyclopentadienone with 1,2-dimethyl-3-carbomethoxycyclopropene did yield the corresponding cycloheptatriene, **47**, containing two methyls, two phenyls and three ester groups. Analogously, a number of cycloheptatrienes bearing multiple electron-withdrawing substituents–esters, nitriles and trifluoromethyl groups, as in **48**, were readily obtainable [58].

## 4. Diels-Alder Cycloadditions en Route to Organometallic Molecular Brakes

### 4.1. Attempted Preparation of Fluorenyl-Pentaphenylbenzene

**Scheme 19:**
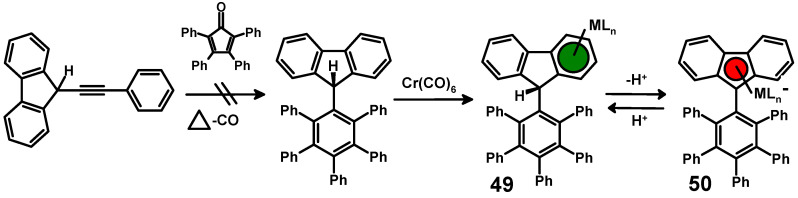
Proposed Diels-Alder synthesis of fluorenyl-pentaphenylbenzene with subsequent incorporation of an organometallic moiety capable of undergoing a haptotropic shift.

In earlier work, we reported that in [C_6_Ph_6_]Cr(CO)_3_, **31**, in which the metal carbonyl tripod is attached to a peripheral ring (Scheme 12), there is a substantial barrier (50 kJ mol^−1^) to rotation of the complexed phenyl substituent relative to the central ring [48]. In light of this observation, we speculated that incorporation of an organometallic moiety onto an external benzo ring of fluorenyl-pentaphenylbenzene could then undergo a base-promoted η^6^ to η^5^ haptotropic shift, as in **49** to **50**, such that the bulky metal carbonyl group would then be sited proximate to the C_6_Ph_5_ core, thereby restricting its rotation and functioning as an organometallic molecular brake (Scheme 19).

**Scheme 20:**
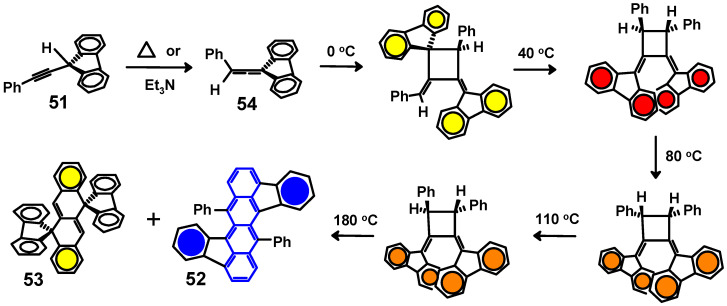
Isomerisation of phenethynylfluorene, **51**, to allene, **54**, dimerisation to form a series of diphenyl-bis(fluorenyliden)cyclobutanes and, ultimately, the tetracenes **52** and **53**.

To this end, we attempted to carry out the Diels-Alder addition of phenethynylfluorene, **51**, to tetracyclone. However, analysis of the products showed complete recovery of the unreacted tetracyclone and formation of two tetracenes, **52** and **53** [59]. Further study revealed that the alkyne had isomerised to the allene, **54**, which dimerised and progressed via a series of highly coloured (yellow, red, orange) bis(fluorenyliden)cyclobutanes before finally forming the tetracenes. Each successive product was characterised spectroscopically and by X-ray crystallography, and the entire sequence depicted in Scheme 20 has been described in detail [60,61]. 

**Figure 17:**
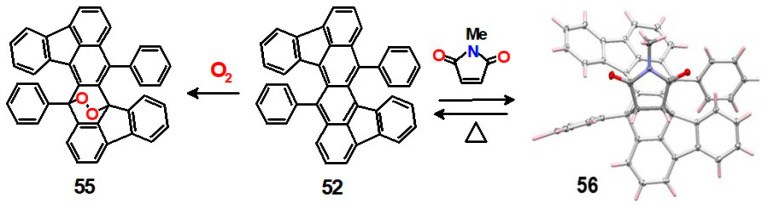
Reactions of tetracene **52** with oxygen to form **55**, and N-methylmaleimide to form **56**.

Nevertheless, we note that the blue tetracene, **52**, which is electroluminescent with potential use in display screens, is susceptible to aerial oxidation and forms the peroxide, **55** [59,60]. Fortunately, the tetracene can be conveniently stored, in a thermally reversible process, as **56**, its Diels-Alder adduct with N-methylmaleimide, whose X-ray crystal structure is shown in Figure 17 [62].

### 4.2. Successful Route to a Molecular Brake via Addition of Benzyne to 9-(2-Indenyl)anthracene

**Scheme 21:**
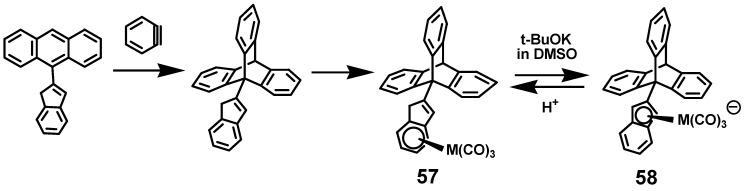
Deprotonation of **57** brings about an η^6^**→**η^5^ haptotropic shift forming **58** in which paddlewheel rotation is dramatically slowed on the NMR time-scale.

More recently, the Diels-Alder cycloaddition of benzyne to 9-(2-indenyl)anthracene to form 9-(2-indenyl)triptycene was crucial in the successful generation of an organometallic molecular brake (Scheme 21). In this case, the η^6^ to η^5^ haptotropic shift (**57**→**58**) of an M(CO)_3_ fragment (M = Cr, Mn or Re) positioned the metal carbonyl tripod so as to block rotation of the triptycene paddlewheel [63]. X-ray data were obtained for **57** and for a neutral version of **58**. This project, including many related Diels-Alder reactions, has been fully discussed elsewhere in this journal [64].

## 5. Diels-Alder Cycloadditions with Subsequent Molecular Rearrangement

**Scheme 22:**
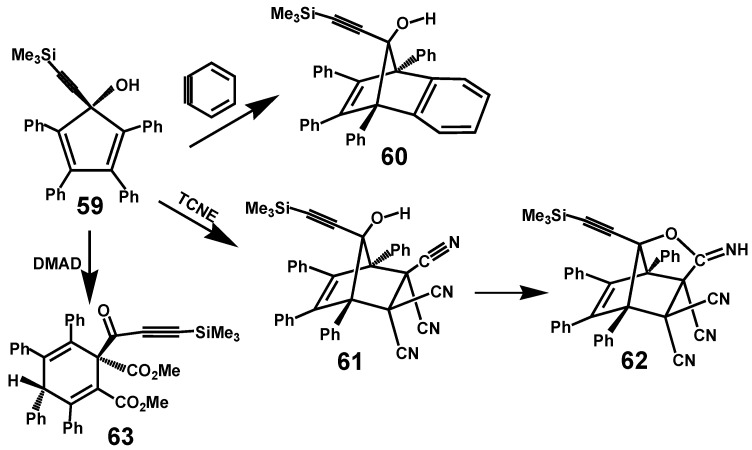
Diels-Alder reactions of 5-trimethylsilylethynyl-1,2,3,4-tetraphenylcyclopentadien-5-ol with benzyne, TCNE and DMAD.

As part of a study of [4 + 2] cycloadditions to 5-trimethylsilylethynyl-1,2,3,4-tetraphenyl-cyclopentadien-5-ol, **59**, this molecule was allowed to react with benzyne, tetracyanoethylene (TCNE) and dimethyl acetylenedicarboxylate (DMAD) [65]. While benzyne yielded the anticipated alkynyl-benzonorbornadienol, **60**, the ^13^C NMR spectrum of the TCNE adduct exhibited resonances at 113.0, 112.1 and 110.5 ppm, indicating the presence of more than the two nitrile environments expected for the mirror symmetric structure, **61**. Moreover, the identity of a peak at 159.4 ppm was not immediately apparent. Fortunately, X-ray crystallography resolved these issues when the product was revealed as the imino lactone, **62**, whereby the hydroxyl moiety was added across an exo-nitrile linkage, thus breaking the mirror symmetry and rendering the other three nitriles nonequivalent (Scheme 22). Evidently, the reaction of TCNE with **59** proceeded via cycloaddition syn to the hydroxyl substituent, in accord with theoretical calculations [66].

**Scheme 23:**
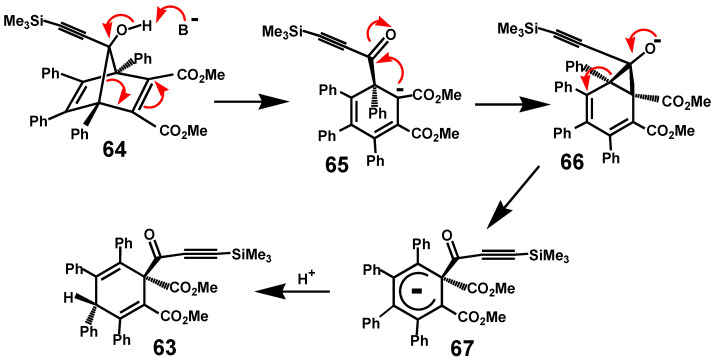
Proposed mechanism for the rearrangement of norbornadiene **64** to cyclohexadiene **63**.

In contrast, the analogous reaction of **59** with DMAD was considerably more complicated, and it is not clear that the identity of the resulting product, **63**, would have been unequivocally determined without recourse to X-ray crystallography. As shown in Scheme 23, one can envisage that base-promoted bridge cleavage in the initial cycloadduct, **64**, followed by intramolecular anionic attack on the newly-formed ketonic functionality in **65**, leads to the cyclopropyl intermediate, **66**. Subsequent regeneration of the ketone with concomitant ring opening completes the migration of the alkynyl ketone moiety to give **67** which, upon protonation, yields the observed product **63** [65].

## 6. Concluding Remarks

In this review, we have attempted to illustrate how the Diels-Alder reaction underpins several projects carried out over a number of years, but not previously gathered together. These include the complete elucidation of the mechanisms of molecular rearrangements whereby the existence of proposed short-lived intermediates, such as isoindenes, can be verified by their interception as [4 + 2] cycloadducts. While the reaction of alkynes with cyclopentadienones has been widely invoked for preparation of arenes, the use of cyclopropenes for the synthesis of sterically crowded seven-membered rings had been far less studied, and the characterisation of such molecules bearing ferrocenyl and/or multiple naphthyl substituents is described herein. 

It is also instructive to note cases where attempted Diels-Alder cycloadditions yielded unexpected products, either through subsequent rearrangement of the initial adducts or, instead, revealed novel chemistry involving a coreactant. This latter situation is exemplified by the serendipitous formation of electroluminescent tetracenes when seeking a route to a molecular machine. Gratifyingly, however, in the first demonstration of an organometallic molecular brake, a benzyne cycloaddition was crucial to its success. Over the past 90 years, the remarkable versatility and applicability of this reaction has attracted the attention of generations of chemists, and we all owe a considerable debt to Otto Diels and Kurt Alder [67].
